# Tumor deposits: markers of poor prognosis in patients with locally advanced rectal cancer following neoadjuvant chemoradiotherapy

**DOI:** 10.18632/oncotarget.6656

**Published:** 2015-12-18

**Authors:** Lu-Ning Zhang, Wei-Wei Xiao, Shao-Yan Xi, Pu-Yun OuYang, Kai-Yun You, Zhi-Fan Zeng, Pei-Rong Ding, Hui-Zhong Zhang, Zhi-Zhong Pan, Rui-Hua Xu, Yuan-Hong Gao

**Affiliations:** ^1^ Department of Radiation Oncology, Sun Yat-sen University Cancer Center, State Key Laboratory of Oncology in South China, Collaborative Innovation Center for Cancer Medicine, Guangzhou, Guangdong, China; ^2^ Department of Colorectal Surgery, Sun Yat-sen University Cancer Center, State Key Laboratory of Oncology in South China, Collaborative Innovation Center for Cancer Medicine, Guangzhou, Guangdong, China; ^3^ Department of Medical Oncology, Sun Yat-sen University Cancer Center, State Key Laboratory of Oncology in South China, Collaborative Innovation Center for Cancer Medicine, Guangzhou, Guangdong, China; ^4^ Department of Pathological Oncology, Sun Yat-sen University Cancer Center, State Key Laboratory of Oncology in South China, Collaborative Innovation Center for Cancer Medicine, Guangzhou, Guangdong, China; ^5^ Department of Oncology, The Second Affiliated Hospital of Sun Yat-sen University, Guangzhou, Guangdong, China

**Keywords:** adjuvant chemotherapy, locally advanced rectal cancer, neoadjuvant chemoradiotherapy, prognosis, tumor deposits

## Abstract

**Background:**

Tumor deposits (TDs) were reported to be poor prognoses in colorectal carcinoma, but the significance in locally advanced rectal cancer (LARC) (T3-4/N+) following neoadjuvant chemoradiotherapy (neo-CRT) and surgery is unclear. Since adjuvant chemotherapy showed no benefit for LARC following neo-CRT, it is of great value to investigate whether TDs can identify the subgroup of patients who may benefit from adjuvant chemotherapy.

**Methods:**

Between 2004 and 2012, 310 LARC patients following neo-CRT and surgery were retrospectively reviewed. Overall survival (OS), disease-free survival (DFS), distant metastasis free survival (DMFS) and local recurrence free survival (LRFS) were evaluated by Kaplan-Meier method, log-rank test and Cox models.

**Results:**

TDs-positive patients showed adverse OS, DFS and DMFS (all *P*≤0.001), but not LRFS (*P* = 0.273). In multivariate analysis, TDs continued to be associated with poor OS (HR = 2.44, 95% CI 1.32-4.4, *P* = 0.004) and DFS (HR = 1.99, 95% CI 1.21-3.27, *P* = 0.007), but not DMFS (HR = 1.77, 95% CI 0.97-3.20, *P* = 0.061) or LRFS (HR = 1.85, 95% CI 0.58-5.85, *P* = 0.298). Among TDs-positive patients, adjuvant chemotherapy significantly improved OS (*P* = 0.045) and DMFS (*P* = 0.026), but not DFS (*P* = 0.127) or LRFS (*P* = 0.862).

**Conclusions:**

TDs are predictive of poor survival in LARC after neo-CRT. Fortunately, TDs-positive patients appear to benefit from adjuvant chemotherapy.

## INTRODUCTION

Although preoperative neoadjuvant chemoradiotherapy (neo-CRT) and total mesorectal excision (TME) significantly reduce the risk of locoregional recurrence and cancer death in locally advanced rectal cancer (LARC) (T3-4/N+) [1-3], about 30% of patients will eventually develop distant metastases [2, 4, 5]. Adjuvant chemotherapy was assumed to prevent distant metastases. Unfortunately, the most recent meta-analysis demonstrated that adjuvant fluorouracil-based chemotherapy does not improve overall survival (OS), disease free survival (DFS) or distant metastasis free survival (DMFS) of LARC following neo-CRT and TME [6]. Certain subgroups of patients are likely to benefit from adjuvant chemotherapy considering the tumor heterogeneity and divergent response to neo-CRT. Thus it is important to identify groups of patients who would benefit from adjuvant treatment after neo-CRT from those who would not.

Tumor deposits are found in the perirectal and mesenteric adipose tissue around rectal adenocarcinomas. Several editions of American Joint Committee on Cancer (AJCC) staging manual have defined tumor deposits. The current seventh edition classifies tumor deposits as follows: the deposit should be in the pericolorectal fat or adjacent mesocolic fat, it should be away from the leading edge of the tumor, there should be no evidence of residual lymph node tissue, and finally the tumor deposit should be within the lymph drainage area of the primary carcinoma.

Previously, several studies [7-10] had reported that tumor deposits were associated with decreased DFS and may identify patients with more aggressive tumors who need aggressive treatment. However, patients recruited in these studies [7-10] did not receive neo-CRT, and tumor deposits were defined and evaluated according to the old criteria which were quite different from the updated standard. The study by Goldstein et al. [9] restricted to patients with T3N+M0 colon adenocarcinomas, which might limit the extensive application of its findings. Moreover, the study by Belt et al. [11] found that tumor deposits defined by the sixth edition criteria increased the risk of developing recurrence in node-negative colorectal cancer patients. Similarly, a recent study [12] also found tumor deposits to be poor prognostic markers among rectal adenocarcinoma patients using the seventh edition criteria. Contradictorily, Song et al. [13] reported that tumor deposits were not prognostic in rectal cancer, and the category N1c in the seventh edition of the AJCC staging system defined by tumor deposits may not be appropriate for patients receiving preoperative neo-CRT. Thus the role of tumor deposits remained controversial in the current treatment mode of neo-CRT followed by TME. More importantly, none of these studies [7-13] investigated the association between tumor deposits and adjuvant chemotherapy.

Therefore, we included 310 LARC patients treated with neo-CRT and TME, to investigate the prognostic effect of tumor deposits and the association with postoperative adjuvant chemotherapy.

## MATERIALS AND METHODS

### Patients

This retrospective study was approved by the Institutional Review Board at Sun Yat-sen University Cancer Center, and individual informed consent was waived given the anonymous analysis of routine data. A total of 376 patients undergoing neo-CRT followed by radical surgery at our center between Oct. 2004 and Dec. 2012 were identified. Rectal carcinoma was clinically diagnosed based on abdominal and pelvic computed tomography (CT), magnetic resonance imaging (MRI) and endorectal ultrasound (ERUS). Other examinations such as complete blood cell count, liver function tests and serum carcinoembryonic antigen (CEA) and carbohydrate antigen 19-9 [CA19-9] levels were also conducted. All patients had biopsy-proven rectal carcinoma.

### Pathological review

**Figure 1:**
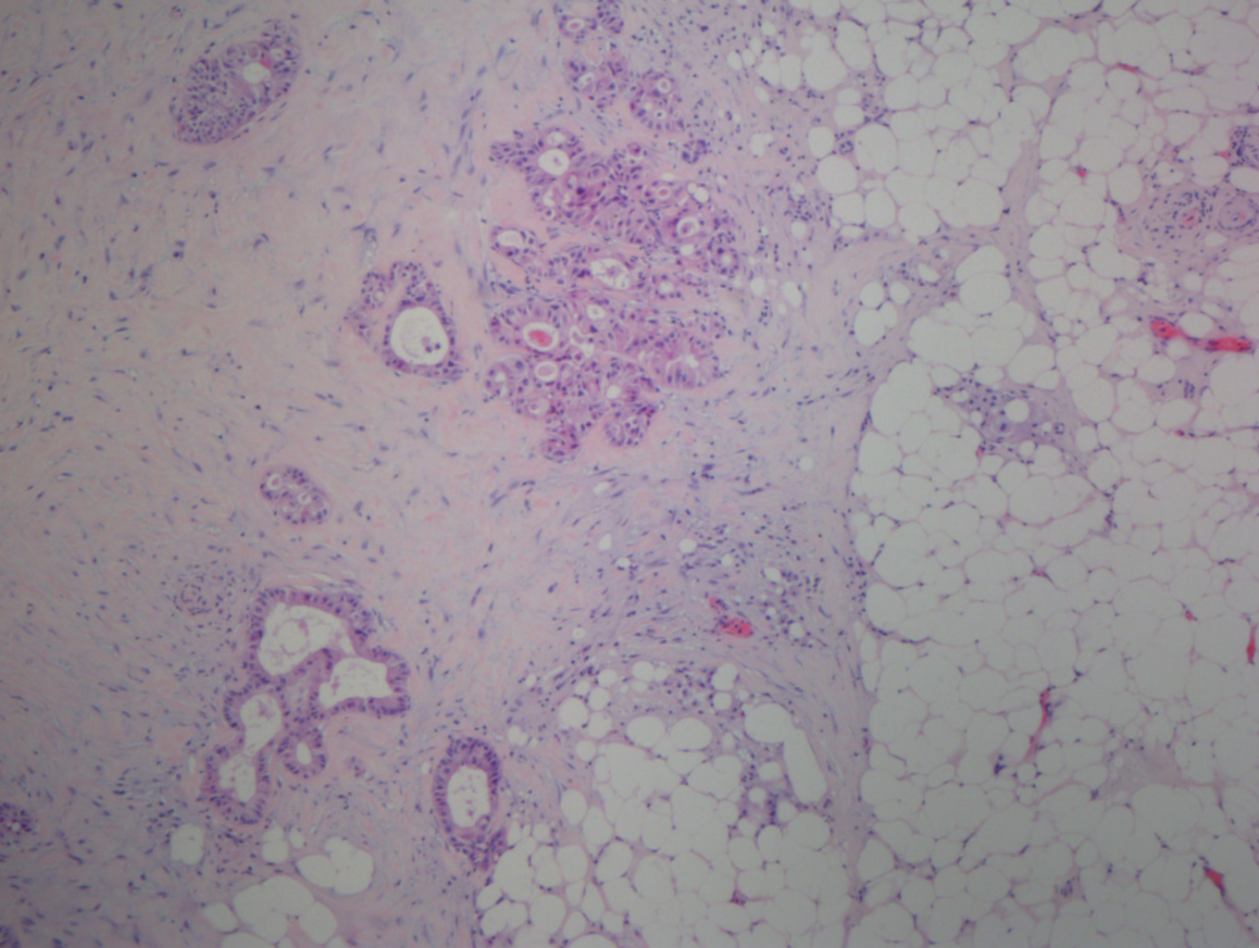
Tumor deposit in rectal adenocarcinoma Tumor deposits in the perirectal fat or adjacent mesocolic fat, they away from the leading edge of the tumor, there should be no evidence of residual lymph node tissue, and the tumor deposits are within the lymph drainage area of the primary carcinoma. (H and E, 40x)

After excluding 21 patients with synchronous distant metastases, another primary malignancy or a prior history of radiotherapy to the pelvis, only 355 patients were eligible. Of these, another 15 resection specimens were missed. In the remaining 340 specimens, 30 specimens were excluded because of the bad stain and quality. Finally, a total of 310 specimens stained with hematoxy-lin and eosin could be used to determine the tumor deposits for the first round by an experienced pathologist (SYX) and the second round by another pathologist (HZZ). They were blinded to the patients’ clinical data and existing pathological outcomes. Tumor deposits were defined and evaluated based on the seventh edition of the AJCC staging manual (Figure 1). Furthermore, tumor regression grading (TRG) classification was evaluated by both pathologists together according to the current AJCC criteria (TRG 0, no residual tumor cells; TRG 1, single cells or small groups of cells; TRG 2, residual cancer with desmoplastic response; and TRG 3, minimal evidence of tumor response).

### Treatment

Radiotherapy was delivered to the whole pelvis at a dose of 46 Gy in 23 fractions, followed by a 4-Gy boost delivered to the primary tumor in 2 fractions for 5 weeks. The radiotherapy technique was based on a three-dimensional conformal radiotherapy treatment planning system (PINNACLE 8) using a 3-field irradiation plan (an 8-MV photon posterior-anterior field and 15-MV photon-opposed lateral beams). The clinical target volume (CTV) included the primary rectal tumor, perirectal tissues, presacral lymph nodes, internal iliac lymph nodes and obturator lymph nodes. For patients with stage T4 cancer or tumors invading the bladder, CTV also included the external iliac lymph drainage area. The superior border of CTV was the bottom of L5, and the inferior border was 2.5-3 cm distal to the tumor. The anterior border was the posterior margin of the bladder or uterus and the posterior border was the anterior margin of the sacrum. The planning target volume (PTV) is defined as the CTV+ 8∼10 mm.

The main preoperative concurrent chemotherapeutic regimens were capecitabine and oxaliplatin (XELOX) or 5-FU, folinic acid and oxaliplatin (FOLFOX6). A total of 259 patients received XELOX (oxaliplatin 100 mg/m^2^, d1+ capecitabine 1000 mg/m^2^ bid, po, d1-14); 42 patients were administered FOLFOX6 (oxaliplatin 85 mg/m^2^, d1+ leucovorin 400 mg/m^2^, d1+ 5-FU 400 mg/m^2^ iv, d1 followed by 2400 mg/m^2^ civ 46-48 h); the remaining nine patients received only Xeloda (capecitabine 1000 mg/m^2^ bid, po, d1-14) due to poor liver or kidney function.

Surgery was performed 6-8 weeks after the completion of preoperative CRT. All patients underwent radical proctectomy, including low anterior resection (LAR), abdominoperineal resection (APR) and Hartmann's procedure.

Postoperative adjuvant chemotherapy was recommended for all patients, irrespective of the surgical pathological results, in accordance with National Comprehensive Cancer Network (NCCN) guidelines. However, only 223 patients actually received adjuvant chemotherapy, either XELOX or FOLFOX6, 4 weeks after surgery. The other 87 patients received no adjuvant chemotherapy owing to postoperative complications, poor overall performance status or economical problem.

### Follow up

Follow up was performed every 3 months for the first 2 years after whole treatment and every 6 months thereafter. Evaluations included complete blood cell count, liver function tests, serum CEA and CA19-9 level tests, physical examination and digital rectal examination at each visit. Chest radiography, abdominal and pelvic CT scanning and colonoscopy were conducted every 6 months after surgery. Positron emission tomography (PET)/CT is not regularly recommended. The last follow up was completed in May 2015.

### Statistical analysis

The primary endpoints were OS and DFS, which were defined as the time from completion of the whole treatment to death from any cause and to the first occurrence of either local or distant progression or of death in the absence of such an event, respectively. The secondary endpoints were DMFS and local recurrence free survival (LRFS). Distant metastasis was identified as any recurrence outside of the pelvic cavity. Local recurrence was defined as any recurrence within the pelvic cavity or perineum.

The balance of covariates among the tumor deposits groups was examined using *t* tests (continuous variables), χ^2^ tests or Fisher's exact tests (categorical variables), as appropriate. OS, DFS, DMFS and LRFS rates were estimated using the Kaplan-Meier method and the log-rank test. Multivariate analysis was performed using the Cox proportional hazards regression. Two-sided *P* < 0.05 was considered statistically significant. All statistical analyses were performed using SPSS software, version 20

## RESULTS

### Patients

The baseline characteristics of the 310 patients were listed in Table 1. Based on ERUS and/or MRI, 27% of patients were diagnosed with clinical stage II disease, and 73% were diagnosed with clinical stage III disease. A total of 75 patients (24%) had a pathological complete response (pCR, ypT0N0M0). The median time interval between CRT completion and surgery was 48 days (range; 20 to 84 days). A total of 186 patients (60%) underwent LAR, 110 (35%) underwent APR, and 14 (5%) underwent Hartmann's procedure. The median follow up was 42 months (range, 5 to 126 months). There were 14 cases (4.5%) of locoregional relapse, 66 cases (21%) of distant metastasis and 62 cases (20%) of death, respectively. Six patients (2%) had both locoregional relapse and distant metastasis. The 3- and 5-year OS rates were 86.7% and 77.2%, and the 3- and 5-year DFS rates were 73.3% and 65.9%, respectively (Table 1).

**Table 1:** Influence of different variables on survival in patients with locally advanced rectal cancer following neoadjuvant chemoradiotherapy

Variables	No.	3-year OS (%)	*P*	3-year DFS (%)	*P*	3-year DMFS (%)	*P*	3-year LRFS (%)	*P*
**Age**			**0.028**		0.425		0.406		0.704
≤55	156	90.0		75.5		82.3		95.8	
>55	154	83.4		71.8		78.0		94.1	
**Sex**			0.515		0.584		0.724		0.262
Male	207	85.6		73.6		80.0		94.3	
Female	103	88.8		73.9		80.4		96.5	
**Tumor location**			0.986		0.821		0.349		0.366
≤5cm	169	85.2		73.6		82.2		93.5	
>5cm	141	88.3		73.6		77.6		96.9	
**CEA (ng/L)**			0.143		0.106		0.099		0.734
≤5	170	90.1		78.5		84.8		94.9	
>5	140	83.5		68.7		75.4		95.4	
**CA19-9 (U/mL)**			**0.003**		**0.001**		**0.039**		0.686
≤35	257	90.0		77.1		82.8		95.0	
>35	53	73.5		57.9		68.2		94.9	
**Tumor grade**			0.769		0.458		0.754		0.978
I	43	92.1		67.9		75.1		97.1	
II	234	87.3		74.9		81.3		94.8	
III	33	81.6		75.5		81.8		93.7	
**Clinical T staging**			0.236		0.633		0.780		0.628
cT2	7	100		85.7		85.7		100	
cT3	131	89.1		74.4		78.6		95.3	
cT4	172	84.5		72.8		81.1		94.6	
**Clinical N staging**			0.286		0.309		**0.026**		0.507
N0	83	87.2		76.9		88.3		95.4	
N1	109	83.9		68.9		72.5		92.3	
N2	118	88.8		75.8		81.2		97.1	
**Clinical stage**			0.148		0.127		**0.019**		0.839
II	84	87.3		77.2		88.5		95.5	
III	226	86.4		72.2		77.0		94.9	
**ypT stage**			**0.004**		**<0.001**		**<0.001**		0.282
ypT0	79	93.0		82.0		92.0		94.0	
ypT1	12	91.8		100		100		100	
ypT2	54	94.3		88.2		94.0		100	
ypT3	140	81.1		62.8		67.9		94.6	
ypT4	25	80.0		64.0		71.2		87.5	
**ypN stage**			**<0.001**		**<0.001**		**<0.001**		0.063
ypN0	233	92.5		80.5		87.1		97.2	
ypN1	60	71.4		54.7		61.0		90.8	
ypN2	17	79.6		47.1		51.3		85.9	
**PCR**			0.086		**0.040**		**0.005**		0.711
No	235	84.8		70.9		76.5		95.0	
Yes	75	92.6		82.4		91.6		95.0	
**AJCC-TRG**			**0.036**		0.066		**0.020**		0.112
TRG0	75	95.5		84.9		92.9		89.0	
TRG1	67	91.5		77.6		81.3		77.0	
TRG2	143	84.8		68.0		74.9		77.2	
TRG3	25	85.7		66.3		71.1		62.0	
**Tumor deposits**			**<0.001**		**<0.001**		**0.001**		0.273
No	256	91.2		78.9		84.0		95.7	
Yes	54	66.4		49.4		61.6		91.9	
**Adjuvant chemotherapy**			**0.026**		0.356		0.636		0.257
No	87	82.7		73.2		80.2		93.4	
Yes	223	88.2		75.9		80.2		95.7	

Note: OS=overall survival; DFS=disease-free survival; DMFS=distant metastasis free survival; LRFS= local recurrence free survival; PCR=pathological complete response; TRG=tumor regression grade.

### Association between tumor deposits and pretreatment and postoperative clinicopathological factors

Overall, elevated pretreatment CEA levels was strongly associated with positive tumor deposits (*P* = .022). Furthermore, postoperative factors, including ypT (*P* < .001) and AJCC-TRG (*P* < .001), were also significantly correlated with tumor deposits. (Table 2).

**Table 2:** Association of tumor deposits with different factors

Characteristic	TD-positive (*n* = 54)		TD-negative (*n* = 256)		*P*
	No.	%	No.	%	
**Age**					0.584
<55	29	53.7	127	49.6	
≥55	25	46.2	129	50.4	
**Sex**					0.985
Male	36	66.7	171	66.8	
Female	18	33.3	85	33.2	
**CEA**					**0.022**
≤5	22	40.7	147	57.4	
>5	32	59.3	109	42.6	
**CA19-9**					0.271
≤35	42	77.8	215	84.0	
>35	12	22.2	41	16.0	
**Clinical T stage**					0.278
T2	0	0	7	2.7	
T3	20	37.0	111	43.4	
T4	34	63.0	138	53.9	
**Clinical N stage**					0.082
N0	9	16.7	74	28.9	
N1	18	33.3	91	35.5	
N2	27	50.0	91	35.5	
**ypT stage**					**<0.001**
ypT0	4	7.4	75	29.3	
ypT1	0	0	12	4.7	
ypT2	8	14.8	46	18.0	
ypT3	33	61.1	107	41.8	
ypT4	9	16.7	16	6.3	
**AJCC-TRG**					**<0.001**
TRG-0	2	3.7	73	28.5	
TRG-1	17	31.5	50	19.5	
TRG-2	29	53.7	114	44.5	
TRG-3	6	11.1	19	7.4	

Note: TRG=tumor regression grade.

### Prognostic effect of tumor deposits in OS and DFS

**Figure 2:**
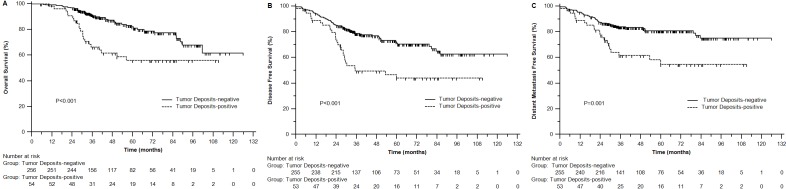
Overall survival (**A**), disease free survival of patients (B) and distant metastasis free survival (C) with different tumor deposits status

Perirectal tumor deposits were detected in 54 of 310 patients (17.4%). In univariate analysis, tumor deposits positive was significantly associated with poor OS (3-year 66.4% *vs* 91.2%, *P* < 0.001), DFS (49.4% *vs* 78.9%, *P* < 0.001) and DMFS (61.6% *vs* 84.0%, *P* = 0.001) (Figure 2A-2C, Table 1).

In addition, we examined the prognostic significance of various clinical and pathological factors. Age (*P =* 0.028), CA19-9 levels (*P* = 0.003), T staging after CRT (ypT) (*P =* 0.004), positive lymph nodes after CRT (ypN) (*P* < 0.001), TRG (*P =* 0.036) and postoperative adjuvant chemotherapy (*P =* 0.026) were all significantly associated with OS. DFS was significantly associated with CA19-9 levels (*P* = .001), ypT (*P <* .001), ypN (*P* < .001) and pCR (*P* = .040). CA-199 levels (*P* = .039), cN (*P* = .026), clinical stage (*P* = .019), ypT (*P <* .001), ypN (*P* < .001), pCR (*P* = .005) and TRG (*P =* .020) were all significantly associated with DMFS. LRFS was marginally correlated with ypN (*P* = .063) (Table 1).

Adjusting for the above significant covariants in the multivariate analysis, tumor deposits continued to be significantly associated with poor OS (HR = 2.44, 95% CI 1.32-4.48; *P* = 0.004) and DFS (HR = 1.99, 95% CI 1.21-3.27; *P* = 0.007). However, tumor deposits did not correlate with DMFS (HR = 1.77, 95% CI 0.97-3.20; *P* = 0.061) or LRFS (HR = 1.85, 95% CI 0.58-5.85; *P* = 0.298) (Table 3).

**Table 3:** Multivariable analysis of different variables on survival in patients with locally advanced rectal cancer following neoadjuvant chemoradiotherapy

Variable	Overall survival		Disease free survival		Distant metastasis free survival		Local recurrence free survival	
	HR (95%CI)	*P*	HR (95%CI)	*P*	HR (95%CI)	*P*	HR (95%CI)	*P*
**Tumor deposits**	2.44 (1.32-4.48)	0.004	1.99 (1.21-3.27)	0.007	1.77 (0.97-3.20)	0.061	1.85(0.58-5.85)	0.298
**CA19-9**	2.91 (1.57-5.40)	0.001	2.20 (1.33-3.65)	0.002	-	-	-	-
**ypT**	-	-	1.24 (1.00-1.54)	0.050	1.59 (1.15-2.19)	0.005	-	-
**Age**	2.00 (1.11-3.63)	0.022	-	-	-	-	-	-

### Adjuvant chemotherapy in subgroup by tumor deposits

**Figure 3:**
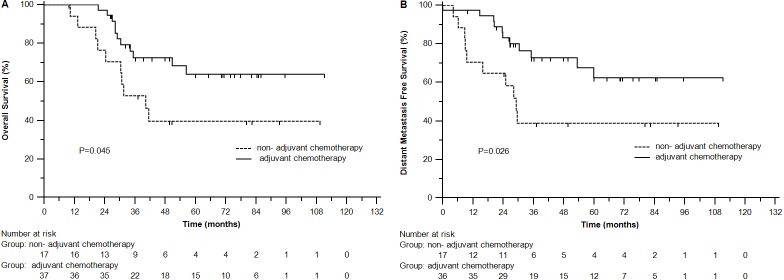
Overall survival (A) and distant metastasis free survival (B) of patients with different tumor deposits status in adjuvant chemotherapy group

In tumor deposits-positive group, adjuvant chemotherapy improved OS (3-year rates 72.6% *vs* 52.9%, *P* = 0.045) and DMFS (72.9% *vs* 38.8%, *P* = 0.026) (Figure 3), but not DFS (*P* = 0.127) or LRFS (*P* = 0.862). In tumor deposits-negative group, there were no significant differences between with and without adjuvant chemotherapy in OS (91.4% *vs* 90.5%, *P* = 0.213), DFS (77.5% *vs* 82.7%, *P* = 0.847), DMFS (81.6% *vs* 90.4%, *P* = 0.065) or LRFS (97% *vs* 94.4%, *P* = 0.073).

## DISCUSSION

In our study, elevated CEA level, advanced ypT and higher AJCC-TRG were associated with tumor deposits in LARC following neo-CRT and TME. Tumor deposits positive patients had poorer OS, DFS and DMFS. Fortunately, postoperative adjuvant chemotherapy appeared to improve the survival of this subgroup of patients.

It is known that elevated CEA level [14], advanced ypT [15] and higher TRG [16] correlate with large tumor size. Considering that patients with tumor deposits after neo-CRT had a TRG of 2 or 3 (minimal or no response) but no TRG of 0 (no viable cancer cells) [12], thus it was not unusual that tumor deposits positive patients in our study had significantly higher pretreatment CEA level, more advanced ypT and higher TRG than those negative ones.

Consistent with prior studies [7-12], we also found that patients with tumor deposits had poorer OS, DFS and DMFS. Importantly, our study had the largest cohort of 310 patients with neo-CRT, using the latest evaluation and definition criteria of tumor deposits. Inversely, Song et al. [13] reviewed 136 ypT3N0M0 rectal cancer, and indicated that tumor deposits showed no prognostic significance. Obviously, the small sample size of this study may lower the confidence of the findings. Given the poor prognostic significance of tumor deposits, we further investigated the role of adjuvant chemotherapy in the according strata. Interestingly, subgroup analysis showed that in tumor deposits-positive group, adjuvant chemotherapy improved OS and DMFS.

The pathogenesis and mechanism of the role of tumor deposits in rectal cancer is unclear at present. Firstly, one pattern of tumor deposits was characterized by microscopic clusters of undifferentiated cancer cells in the fatty tissues, which were morphologically similar to the tumor budding at the invasive front of the main tumor (the socalled peri-tumoral budding) [7]. Researches indicated that peri-tumoral budding was strongly associated with lymphatic invasion and lymph nodes metastasis [17, 18]. And numerious studies [19-24] had confirmed the poor prognostic effect of peri-tumoral budding in colorectal cancer. As tumor budding indicates the early phase of invasion, tumor deposits may represent more vigorous tumor progression. Thus it is reasonable to observe a similar poor prognostic of tumor deposits in our study. Secondly, tumor deposits may acted as satellites of the main tumor to increase the field of invasion, as tumor deposits may be lymph nodes completely replaced by tumor according to AJCC Manual for staging of cancer. In addition, Prabhudesai et al. [10] found a significant association between tumor deposits and vascular invasion, which suggested that a proportion of tumor deposits may represent blood-borne spread and that tumor deposits were early form of metastatic disease in patients with rectal cancer. These highly supported tumor deposits to be a poor prognostic factor as we found. Especially, tumor deposits positive patients were mostly found to be those with advanced ypT stage in the current study. Thus the subgroup of tumor deposits positive patients were very likely to benefit from adjuvant chemotherapy, since adjuvant chemotherapy could not improve the survival of ypT0-2N0 patients but significantly decreased the risk of distant metastasis in ypT3-4N0 patients [25]. And due to the strong association of positive tumor deposits with elevated CEA level and higher AJCC-TRG, besides advanced ypT stage, tumor deposits positive patients after neo-CRT may be a subgroup with poorer prognosis than ypT3-4N0 alone, and consequently obtained more benefit in DMFS and OS from the additional adjuvant chemotherapy.

Of note, Rogers et al. [26] indicated that pretreatment intra-tumoral budding in the rectal biopsies predicted a poor pathological response to neo-CRT. But importantly, tumor budding within the entire tumor is termed intra-tumoral budding [27], which was not the same as the peri-tumoral budding after neo-CRT in our study. The finding in that study by Rogers et al. [26] cannot indicate a similarly poor response to adjuvant chemotherapy after surgery for patients with tumor deposits or peri-tumoral budding in resected specimens. Similarly, in the study by Kim et al. [28], tumor budding-positivity was found to be a significant predictor of poor survival in patients receiving non-oxaliplatin-based adjuvant chemotherapy. But the prognostic impact did not remain in multivariate analysis on one hand; on the other hand, this cannot suggest the absence of benefit from adjuvant chemotherapy in tumor budding (deposits) positive patients.

The main limitation of this study is that the two pathologists did not evaluate the resection specimens independently, which may increase the error of evaluating tumor deposits. And the possibility of confounders and issues with missing data are unavoidable due to the retrospective design. But clinicopathologic and survival data were verified by review of individual patient record. All included patients received standard management of neoadjuvant chemotherapy and TME as recommended.

Overall, this study indicated tumor deposits following neo-CRT to be poor prognostic factors, and found survival benefit from postoperative adjuvant chemotherapy in LARC patients with tumor deposits.
